# Ultrasound‐assisted emerging technologies for chemical processes

**DOI:** 10.1002/jctb.5555

**Published:** 2018-02-28

**Authors:** Anton A Kiss, Rob Geertman, Matthias Wierschem, Mirko Skiborowski, Bjorn Gielen, Jeroen Jordens, Jinu J John, Tom Van Gerven

**Affiliations:** ^1^ School of Chemical Engineering and Analytical Science The University of Manchester Manchester UK; ^2^ Faculty of Science and Technology University of Twente Enschede The Netherlands; ^3^ Janssen Pharmaceutical Companies of Johnson & Johnson Janssen Research & Development Beerse Belgium; ^4^ Laboratory of Fluid Separations TU Dortmund University Dortmund Germany; ^5^ Department of Chemical Engineering KU Leuven, Leuven Belgium

**Keywords:** ultrasound, emerging technologies, industrial applications, roadmap

## Abstract

The chemical industry has witnessed many important developments during past decades largely enabled by process intensification techniques. Some of them are already proven at commercial scale (e.g. reactive distillation) while others (e.g. ultrasound‐assisted extraction/crystallization/reaction) are on their way to becoming the next‐generation technologies. This article focuses on the advances of ultrasound (US)‐assisted technologies that could lead in the near future to significant improvements in commercial activities. The aim is to provide an authoritative discussion on US‐assisted technologies that are currently emerging from the research environment into the chemical industry, as well as give an overview of the current state‐of‐the‐art applications of US in chemical processing (e.g. enzymatic reactive distillation, crystallization of API). Sufficient information is included to allow the assessment of US‐assisted technologies and the challenges for implementation, as well as their potential for commercial applications. © 2017 The Authors. *Journal of Chemical Technology & Biotechnology* published by John Wiley & Sons Ltd on behalf of Society of Chemical Industry.

## INTRODUCTION

In ultrasound (US)‐assisted technologies, the US waves propagate through a liquid with a wavelength that is significantly longer than that of the bond length between atoms in the molecule hence they cannot affect the vibrational energy of the bond, nor directly increase the internal energy of a molecule. However, the enhancing effect of US arises from acoustic cavitation, namely the formation, growth, and implosive collapse of bubbles in a liquid. For example, sonochemistry deals with understanding the effect of US in forming acoustic cavitation in liquids, resulting in the initiation or enhancement of the chemical activity in the solution. Many benefits of using US in other operations (e.g. liquid–liquid extraction, crystallization, reactive distillation) were identified: high energy density source, operational flexibility, fast response time to inlet variations, and low operating costs. However, due to the technical and operational limitations on scale‐up of US systems, the transition from the lab to industrial scale has been hindered so far.

US application has been reported to benefit chemical processes as a result of the various mechano‐chemical effects associated with cavitation induced in liquids. These effects are not limited to homogenous liquid systems but also applicable to heterogeneous systems of liquid–liquid (e.g. solvent extraction by improved interfacial area for mass transfer), liquid–solid (e.g. crystallization by sono‐fragmentation) and liquid–gas/vapor (e.g. reactive distillation) systems. These improvements contribute to process intensification having a significant effect on the feasibility and economic performance of reactive systems. Currently, the use of US in industry is mainly limited due to inefficient US generation and high operating costs, although it is a promising technology not solely for high‐value chemicals and pharmaceuticals, but also in the food industry.^1^, ^2^, ^3^, ^4^, ^5^ The sono‐chemical potential was reported to have beneficial effects for several US‐assisted technologies such as: extraction, distillation and crystallization.^5^, ^6^, ^7^ For example, a number of new US‐assisted extraction processing approaches have been proposed, including the modification of plant cell material to provide improved bioavailability of micro‐nutrients; simultaneous extraction and encapsulation; quenching of the radical sonochemistry to avoid degradation of bioactives; and the use of the radical sonochemistry to achieve targeted hydroxylation to increase bioactivity.^4^ The main benefits reported are the decrease of extraction and processing time, and of the amount of energy and solvents used.^5^ In the case of distillation, an effect of ultrasonic frequency and intensity on the relative volatility and azeotropic composition was claimed and reported.^6^ Applying sonication during crystallization can reduce the average crystal size, allow a uniform particle size distribution (PSD), reduce the induction time, increase the yield and selectivity towards the desired crystal morphology.^7^

However, in order to efficiently utilize the benefits of US, a deep understanding of the exact effects of US on the reaction rates and physical phenomena is required. Therefore, the following points need to be addressed in the knowledge generation: equipment design, scale‐up, field uniformity, and penetration depths.

This perspective paper is the first one to describe the main ultrasound‐assisted emerging technologies for chemical processes – by using highlights of the EU project ALTEREGO – with a focus on the US technologies that were investigated: extraction, crystallization, and reactive distillation. In addition, a novel roadmap for industrial implementation of these US‐assisted technologies is also proposed.

## US‐ASSISTED TECHNOLOGIES

Alternative energy forms for green chemistry (ALTEREGO) was a European project funded within the 7th Framework Programme (theme NMP.2012.3.0‐1) which aimed at overcoming the existing bottlenecks towards implementation of alternative energy technologies for intensified chemical manufacturing (http://www.alterego-project.eu). The consortium composed of eight academic and industrial partners from five European countries planned to establish a new methodology towards enabling highly efficient chemical synthesis with alternative energy forms through reliable process data collection with advanced analytical tools, robust multi‐scale modeling and design and development of scalable equipment. The US technologies were applied to different industrially relevant case studies related to green fuels, bulk chemicals and advanced pharmaceutical synthesis.

### US‐assisted extraction

**Figure 1:**
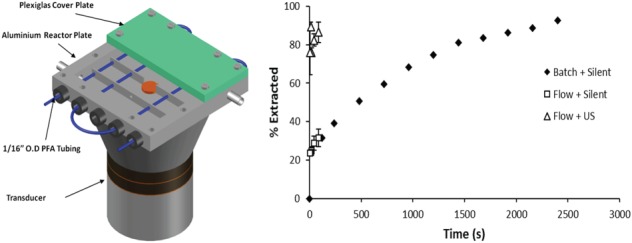
Temperature controlled interval contact reactor (left). Variation of % extracted with residence time for batch – silent condition, flow silent and sonicated experiment (right).

Solvent extraction is a separation process that is commonly used in a wide variety of chemical processes ranging from API manufacturing to nuclear fuel processing. It involves the transfer of solute between two immiscible liquid phases. The challenge here is to provide proper mixing to facilitate sufficient contact between the two immiscible phases for effective mass transfer. The combination of microchannel with ultrasound tackles this by providing a successive improvement of the interfacial area by virtue of the characteristic size of a microchannel and the emulsification by ultrasound. To bring about an effective combination of the ultrasound with a microchannel, a temperature controlled interval contact reactor was designed in the ALTEREGO project (Fig. 1, left).^8^ This design makes use of a solid interface at regular interval between microchannel tubing and ultrasound transducer for effective transfer of power and a liquid medium flowing between the solid interfaces for proper control of the temperature of the reaction mass.^9^

This design was proved to be effective using a model reactive extraction process the hydrolysis of p‐nitrophenyl acetate. In terms of advantages, it provides a low power (input electric power < 30 W), solvent resistant (PFA is the material of choice for the micro‐channel) and contamination‐free (no direct contact of the transducer with the reaction mass) design.

To understand its usability in actual chemical processing, another industrially‐relevant process was selected which is the removal of acetic anhydride from an organic stream with water. The organic phase contains the acetic anhydride dissolved in toluene at a concentration of 0.3 mol L^‐1^. The aqueous phase is distilled water. The two phases are pumped through a T‐junction into the designed reactor maintained at a temperature of 25 °C and the mixed reaction mass is separated in a centrifuge at 2700 rpm for 2 min. The ultrasound experiments were conducted at a frequency of 42 kHz, amplitude of 590 mV and net applied power of 20 W. The final aqueous and organic streams were analyzed with an FTIR probe. The results obtained were compared with a batch experiment performed under silent condition at the same temperature (Fig. 1, right). The results show similar extraction yields for the silent condition in batch and flow, but sonication resulted in much faster extraction, with respect to time required for attaining the same yields: e.g. the time was reduced by a factor of 22 to attain an extraction of 86%.

### US‐assisted crystallization

The use of US in crystallization processes has been extensively studied.^10^, ^11^, ^12^, ^13^, ^14^ Initially, the main focus has been on ultrasonic seeding, but it has also been shown that application of ultrasound affects crystal growth kinetics and also influences (de)agglomeration. The use of ultrasound as replacement for classic seeding in a batch process is attractive. The classic seeding approach requires seed addition at a fixed point in the process, usually halfway in the metastable zone. In practice this is a cumbersome process; seeds often have to be specially prepared, e.g. by micronization, enough seed material should be reserved for the batch or the campaign and addition of seeds at large scale is laborious.

In situ seeding of ice crystals using ultrasound is also of interest for the food industry.^15^ For obvious reasons it is impossible to add seeds during freezing of food stuffs. Generating ice crystals by US during freezing of foodstuffs leads to smaller ice crystals and consequently less damage to the cellular structure and better preservation.

Changing the morphology of crystals using ultrasound is also of interest; especially for high dose APIs the crystal morphology is an important factor in the formulation development. Also in the food industry crystal morphology is important; the shape of ice crystals can influence the organoleptic sensation of ice‐cream significantly.

That being said there are still some hurdles to overcome. First of all equipment for large‐scale application should become available. Second, there should be an incentive to implement the technology. In contrast to the fine and bulk chemical industry, time to market considerations often override considerations like lower energy usage. Emphasis from the pharmaceutical perspective should be more on utilizing the potential of US‐assisted crystallization in terms of speed of development and ease of scaling up rather than the lower energy consumption. From the food industry perspective time to market is less important, but also here scale‐up and equipment issues are clearly identified. This is also reflected in the road map discussed in the following sections.

#### Pulsed ultrasound

Industrial crystallization often uses seed crystals to induce nucleation in a reproducible way, avoiding stochastic behaviour which could lead to batch‐to‐batch variations. As a result, crystals within a desired size range and a narrow span are obtained. Sonication was proven to be a suitable alternative for this conventional approach as the seeds can be generated in situ by the presence of acoustic cavitation bubbles and accompanying effects. However, the additional energy requirement of US could inhibit scale‐up and industrial application.^16^, ^17^ Therefore, the use of pulsed sonication during crystallization processes is recommended as it reduces the energy demand by periodically turning the sound field on and off.^18^

**Figure 2:**
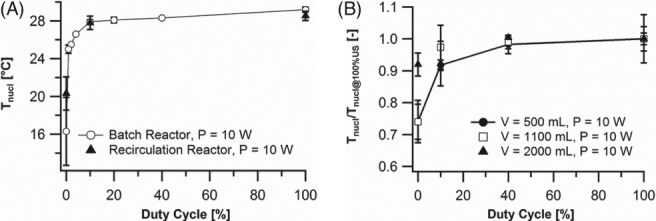
Effect of different ultrasonic pulse settings on nucleation temperature of paracetamol. A duty cycle of 0% corresponds to silent conditions. (left) Comparison between batch and recycled flow crystallizer with a volume of 250 mL, (right) evaluation of scalability in batch reactors with various volumes.

On the one hand, it was reported that the minimal ultrasonic pulse setting or corresponding power threshold to improve nucleation depends on the supersaturation level and compound. Due to lack of data of various crystallization systems, this will require an experimental approach to minimize the ultrasonic energy use.^19^ On the other hand, theoretical bubble dissolution calculations can be employed to determine the minimal duty cycle of the ultrasonic field.^20^ The latter method was used in the ALTEREGO project during ultrasound‐induced nucleation of paracetamol under pulsed sonication at 30 kHz, suggesting a minimal duty cycle in the range of 9–13%.^21^ Next, these calculations were experimentally validated by determination of the nucleation temperature of paracetamol during application of pulsed ultrasound with various duty cycles in a batch and recirculation reactor. Figure 2 (left) shows the results of these experiments and confirms that a duty cycle of about 10% provides a similar improvement in nucleation to that of continuous sonication. Furthermore, Fig. 2 (right) indicates that this methodology is even scalable to some extent, since batch experiments with various volumes again exhibit a threshold behavior at a duty cycle of 10%. However, at 2000 mL, ultrasound no longer improves the nucleation temperature, suggesting that a higher ultrasonic power is required when the reactor volume is increased significantly.^16^, ^22^

Overall, this research indicates that pulsed US, optimized by bubble dissolution calculations, can significantly increase the nucleation temperature. Although additional energy is required to power the ultrasonic actuator, the periodic use of US reduces the energy demand by ∼90% compared with continuous operation, without a significant effect on the nucleation temperature. Pulsed ultrasonic technology could be applied to generate nuclei in crystallization processes which cannot be seeded by the conventional approach (e.g. sterile production).

#### Optimal ultrasound parameters for industrial application

**Figure 3:**
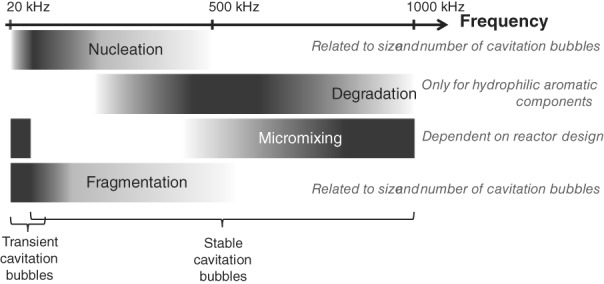
Overview of optimal frequencies for nucleation, degradation, micromixing and fragmentation. Dark colors indicate elevated nucleation, degradation, micromixing or fragmentation rates.

Most commercial available equipment is designed for cleaning applications or high shear mixing.^23^ These off‐the‐shelf ultrasonic probes or cleaning baths work at low ultrasound frequencies of 20 to 40 kHz. Low frequencies generate large cavitation bubbles which will implode violently and hence generate high shear and create microjets which are able to clean surfaces. However, US is used for a lot more than just cleaning or high shear mixing and for some applications other frequencies are more appropriate. Figure 3 summarizes the optimal ultrasonic frequencies for nucleation, degradation, micromixing and fragmentation, as studied in the ALTEREGO project.^24^

The low frequency range of 20 to 40 kHz seems optimal to generate higher nucleation and fragmentation rates. However, the exact optimal frequency is probably reactor and system specific. Kaur Bhangu et al. 25 for example, investigated the antisolvent crystallization of paracetamol under different ultrasonic frequencies of 22, 44, 98 and 139 kHz and found the lowest induction times at 98 kHz. They speculated that, at 98 kHz, there was an optimum between the number of cavitation bubbles and the shear magnitude generated by the bubbles. The number of bubbles is, however, influenced by the number of antinodes present in the vessel which depends mainly on the applied frequency, the solvent and reactor design.

High ultrasound frequencies around 1 MHz are favorable for processes which require good micromixing such as anti‐solvent crystallization, neutralization, combustion or polymerization reactions. The same levels of micromixing can also be achieved at low frequencies around 20 kHz and transient cavitation bubbles. However, these transient cavitation bubbles implode very violently so that they can induce breakage of solid products and induce defects in the particles. It is less clear whether the specific optimal frequency to enhance micromixing is independent of the reactor design or not.

Besides enhancements of nucleation, fragmentation and micromixing, ultrasound has also the potential to induce degradation reactions and hence significantly impact the final purity. This is particularly important for the pharmaceutical industry where impurities are detrimental for the final product quality. Degradation of hydrophilic aromatic components occurs mainly at the intermediate frequencies of 200 to 800 kHz. ·OH radicals and H_2_O_2_ are produced which cause radical degradation reactions with the hydrophilic aromatic components which are present near the bubble wall.^26^ In contrast, volatile aromatics degrade mainly via pyrolytic degradation and show higher degradation rates at low US frequencies around 20 kHz.^27^

### US‐assisted reactive distillation

Reactive distillation (RD) can offer key techno‐economic advantages over a sequential combination of reaction and separation (e.g. high conversion and selectivity, low investment and operating costs) making it suitable for successful production.^28^, ^29^ However, rather slow reactions might need to be accelerated in order to be transferable into RD. Ultrasound has been shown to be able to accelerate the reaction rates of electrochemical, chemical and enzyme‐catalyzed reactions.^30^, ^31^ The possible implementation requirements of alternative energy forms (as US) in RD were recently shown on the basis of the enzyme‐catalyzed transesterification of ethyl butyrate.^32^ The combination of US, RD and enzyme‐catalyzed reactions leads to synergies, which can improve process economics in the case of high reaction rate improvement. Nevertheless, several measures are necessary before industrial implementation, which are discussed hereafter.

Similar to chemically catalyzed reactions, in situ separation can also provide benefits for bio‐catalyzed reactions, such as by applying enzymes in an integrated reactive distillation process, the so‐called enzymatic reactive distillation (ERD), which can overcome equilibrium limitations and provide a cost advantage over classic batch processes. One possibility to further intensify ERD is the use of alternative energy forms, such as the application of ultrasound (US) in order to enhance enzymatic reactions by increasing the diffusion rates to the active sites of the enzymes or the enzymes' activity itself.^33^, ^34^ An enhancement in enzymatic reaction rates by factors of up to ten was recently demonstrated by means of the application of US for exemplary reaction systems.^30^, ^35^ The combination of US and ERD to form a US‐assisted ERD (US‐ERD) consequently offers the potential to exploit further benefits. However, the extent to which these synergies can be exploited depends strongly on the chemical system and the enzymes utilized. The current section illustrates how to evaluate the potential benefits of this innovative process concept by providing an overview on the characterization of reaction kinetics with and without US, experimental ERD investigations, as well as modeling and optimization of ERD and US‐ERD processes. The specific case study used here is the transesterification of ethyl butyrate with Candida antarctica lipase B (CalB) to produce butyl butyrate, which is a volatile ester used in the flavor industry^36^:


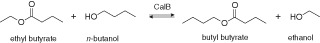


**Figure 4:**
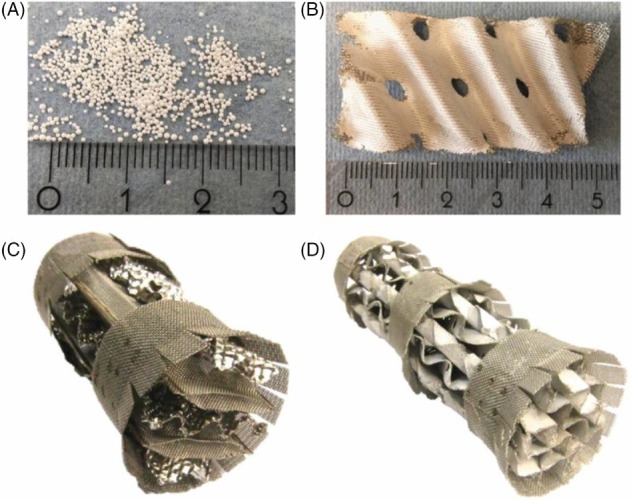
(a) Enzyme beads in Katapak‐SP^®^, (b) coated sheet section, (c) coated packing element and the whole packing (d).

In order to account for the limited stability of most enzymes concerning temperature and pH, immobilization of the enzymes in the column is of significant importance to protect them from the elevated temperatures in the reboiler. Furthermore, as in every RD column, the immobilization offers the potential to implement a separation section in the column in which no catalyst is present. Two possible types of catalytic packings that enable the immobilization of enzymes are the surface coating of porous spherical acrylic polymer resins (termed enzyme beads) which are further filled into a catalytic packing like Katapak‐SP^®^, while the other type is an entrapment of enzymes in a silica‐gel based coating that is applied onto a structured packing like Sulzer BX^®^ (as shown in Fig. 4).

Both types of immobilization (enzyme beads and coated packing) were evaluated in the ALTEREGO project and showed stable performance during investigation of the reaction performance without US building the basis for ERD application. However, when introducing US irradiation the enzyme beads were almost fully disintegrated, ruling out this combination for the given system.^37^ However, Martins *et al*.^38^ observed enhanced operational stability of enzyme beads under US irradiation compared with mechanical agitation for the esterification of acetic acid and *n*‐butanol. Since this result was validated by Wierschem *et al*.^37^ as well, it can be concluded that the enzyme bead stability is strongly dependent on the applied chemical system and the feasibility of any combination of immobilization and US irradiation needs justification prior to implementation for a specific chemical system. Furthermore, it was observed that low‐frequency US rather destroys the immobilizate compared with high‐frequency US, which is confirmed by Ajmal *et al*.^39^

Consequently, both enzyme beads and coated packing are suitable solutions for the transesterification of ethyl butyrate with CalB, while for US‐ERD only the coated packing provides a feasible option. As a basis for further model‐based evaluation and process design reaction kinetic models were derived for the different types of immobilizates and the combination with US for the coated packing. Being based on a specific ordered Ping‐Pong Bi‐Bi kinetic approach, the kinetic models showed excellent agreement with the experimental results.^37^

The next step in demonstrating the feasibility of an ERD process is the implementation in a pilot‐scale experimental investigation of the ERD with enzyme beads and coated packing. The successful implementation was demonstrated by Wierschem *et al*.^40^ in a glass‐made distillation column that features a packing height of more than 5 m and DN50/51 mm inner diameter. The experiments demonstrated the feasibility of the process including a negligible loss of activity of the enzymes during 200 h of operation.

As a final step to build the foundation for an evaluation of the potential of ERD an accurate model has to be developed and validated against the experimental results obtained from the pilot‐scale ERD experiments. Because of the strong integration of the different kinetic phenomena a rate‐based model is required, building on the MERSHQ equations (mass and energy balances, rate expressions, summation constraints, hydraulic equations and interfacial eQuilibrium conditions), in combination with accurate physical property data and correlations describing the hydrodynamics and mass transfer kinetics for the applied equipment. A similar model for the transesterification reaction and the experimental data was developed in the Aspen Custom Modeler^®^ environment and validated against the experimental data by Wierschem *et al*.^40^

Based on the developed model a model‐based investigation can be performed in order to analyze the potential benefits of an optimization‐based design study for a certain production scenario. Wierschem *et al*.^32^ performed such an analysis for an industrial‐scale ERD and US‐ERD process, considering the synthesis of 10 kilotons of butyl butyrate per year with a desired purity of 99 wt%. Both processes were optimized in terms of minimization of the annual costs by using an evolutionary algorithm in combination with the simulation model. The techno‐economic evaluation showed that the benefits from US application concerning the intensification of the reaction rates in the US‐ERD process is approximately compensated by the additional costs for transducers, such that ERD and US‐ERD processes result in approximately equal costs. While the installation costs of the US equipment are high, they are compensated by a 12% lower reactive section height and a 7% lower total height of the US‐ERD column in comparison with ERD. Taking into account that significantly larger improvements of reaction rates were reported for other enzymatic reaction systems,^30^, ^35^, ^38^ a sensitivity study was performed concerning the reaction rate improvement. A further increase of conversion above the 50% (which were experimentally determined for this system) would offer significant cost advantages.

## ROADMAP

Despite the significant progress in research over recent years, ultrasound technology in its current state is not ready for large‐scale industrial application. Apart from the need for more basic understanding of the process, a common denominator in the different application areas is the need for scale‐up. Having solved these issues the economic evaluations can be made, identifying the areas where application of US technologies would show an added benefit. To structure the efforts needed for successful introduction of US technologies a number of roadmaps have been set up for the different application areas. Based on the academic research, the industrial companies involved in the ALTEREGO project developed a roadmap for implementation of two of the three investigated technologies (as the third one would be very similar), namely US crystallization and US‐ERD.

### US‐assisted crystallization

The roadmap for implementing US‐assisted crystallization consists of key phases, specifically tailored towards the pharmaceutical industry, and contains a number of elements only relevant to this industry. For the fine chemicals industry these specific elements may be omitted.

- Phase 1: identification of application areas and freedom to operate. The following areas have been identified for US‐assisted crystallization: batch crystallization (seeding, milling, morphology control), continuous crystallization and sterile crystallization. The last application is particularly attractive as no easy solution is available for sterile seed addition.
- Phase 2: addressing remaining fundamental questions. These concern the following areas: control of the polymorphic form, tip erosion, radical formation in certain solvents (e.g. THF), scale‐up strategy (hardware and scale‐up rules)
- Phase 3: assessing regulatory impact and defining an introduction strategy. For application in the final API step the regular steps need to be taken. From a pharmaceutical standpoint US‐assisted crystallization is simply another technology, nevertheless critical process parameters, process ranges, etc. need to be identified.
- Phase 4: build good‐manufacturing‐practice (GMP) pilot equipment. For the successful introduction of the technology the availability of GMP qualified equipment at risk is vital. Timelines for the development of new drugs are short and development projects cannot wait for building, testing and qualifying equipment in a GMP setting.
- Phase 5: identifying a ‘launch’ product for the technology. If a project can be found where application of ultrasound adds a clear benefit, the need for this technology would override most budget and time constraints. A drawback is that the timelines will be governed by the project timelines rather than the progress of the development of the US technology.
- Phase 6: introduction in production. Based on the experience obtained with the GMP pilot plant a selected production site will be equipped with US equipment.

Introduction of US‐assisted crystallization will take a significant period of time – in the most optimistic scenario 8 years but 10–15 years is a more realistic estimation (European Road Map for PI Technology, Process intensification ‐ Chemical Sector Focus Technology Assessment).

### US‐assisted reactive distillation

**Figure 5:**
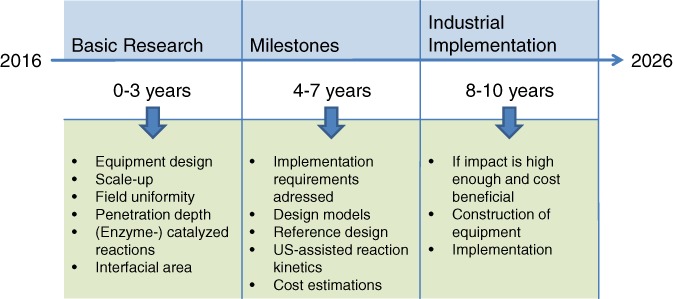
Timeline for the implementation of US‐assisted RD in industry.

Techno economic evaluations as presented by Wierschem *et al*.^32^ are indispensable in order to analyze if the tremendous improvements of the reaction rates by US application reported in literature,^30^, ^31^, ^35^ can be sufficiently exploited in US‐assisted RD/ERD. For processes with a sufficient economic potential process control needs to be established to adjust process conditions automatically and compensate for any feed fluctuations. The open issues that need to be investigated in future work in order to establish US‐ERD/US‐RD in industry are summarized in Fig. 5.

The roadmap for implementing US‐RD / US‐ERD consists of several phases:

- Phase 1: identification of application areas. US‐RD / US‐ERD application could be used to produce components such as amines, amides, alcohols, oxiranes, esters and epoxides that could be enantiomerically pure either for pharmaceutical, chemical or food applications.
- Phase 2: addressing remaining fundamental questions and scale‐up strategies as discussed in the section ‘Discussion’. Since US field generation and propagation in industrial‐scale applications as well as construction of US equipment for RD is hardly examined, US‐RD/US‐ERD lacks practical examples.
- Phase 3: assessing regulatory impact and defining an introduction strategy. US application in RD/ERD is a new field where the process engineer needs to evaluate how regulations in RD applications change by applying US concerning, for example, noise control or plant safety.
- Phase 4: build pilot equipment at a relevant scale. As examples of laboratory or pilot‐scale equipment of US‐RD / US‐ERD are missing in this phase, first studies should provide applicable equipment including construction and operation knowledge.
- Phase 5: identifying a specific product for which the technology is beneficial. As the techno economic evaluation presented by Wierschem *et al*. 32 shows a significant increase in the reaction rate by US application compared with the unsonicated process does not necessarily lead to cost savings. Therefore, a clear benefit of US application needs to be found prior to its use.
- Phase 6: introduction in production at large scale. As presented in Fig. 5 industrial implementation of US technology in RD/ERD could not be implemented tomorrow. A sophisticated approach including design models, scale‐up strategies and more needs to be followed before an industrial implementation.

## DISCUSSION

Although several positive effects of ultrasound on crystallization processes are reported in the literature, further developments are required before industrial implementation is feasible. Clearly some fundamental questions should be solved. In this research it was shown that the optimal ultrasound frequency for nucleation and sono‐fragmentation lies around 40 kHz. However, this frequency is probably influenced by the number and size of the cavitation bubbles and hence depends on the applied frequency, the reactor geometry and the choice of the solvent.^25^ In addition, the mechanism behind ultrasound‐assisted nucleation is not known today. Several hypotheses exist to explain the effect of ultrasound on nucleation.^41^, ^42^, ^43^ These hypotheses relate the effects of ultrasound to the size of the bubbles, the speed of the bubble implosions or the flows introduced by the cavitation bubbles. All these effects are optimal at low ultrasonic frequencies between 20 and 100 kHz, but the exact optimal ultrasound parameters can differ between the different effects. Therefore, more research is needed to unravel the exact mechanism so that efficient ultrasound crystallizers can be designed more optimally.

Besides speeding up nucleation, ultrasound has also the potential to significantly impact the final crystal purity. Degradation of pharmaceutical compounds can result in impurities which are detrimental to the final product quality of pharmaceutical products. During this research, it was shown that paracetamol degradation occurs mainly at intermediate frequencies between 165 and 850 kHz. However, this frequency range is only valid for hydrophilic aromatic components. These components show predominantly the same degradation mechanism as paracetamol, namely degradation via radical chain reactions at the bubble–solution interface.^44^ Nevertheless, volatile aromatics degrade mainly via pyrolytic degradation and show therefore higher degradation rates at low ultrasound frequencies around 20 kHz.^27^ It is nonetheless not clear whether this pyrolytic degradation occurs for pharma components as well and how radical production is impacted by the solvent.

Besides fundamental issues, research is also needed on the efficient application of ultrasound. In this research it was shown that pulsed ultrasound can create similar effects to continuous sonication but with a 90% lower energy consumption. This energy consumption of ultrasound should be as efficient as possible to make industrial application of ultrasound feasible. Pulsation is therefore a viable option. The question is, however, how pulsation should be applied in a flow system and whether it has the same beneficial effect on sono‐fragmentation or micromixing. Also, efficient scaling up strategies for ultrasound should be investigated. For the pharmaceutical industry in particular, scaling up by introducing larger ultrasound probes is an unrealistic option. On one hand these larger probes will create a more inhomogeneous ultrasound field which can result in more batch‐to‐batch variability. On the other hand, erosion of the probe tip can introduce unwanted contamination of the products which can be detrimental to product quality.

Before an industrial implementation of US‐ERD further developments are necessary. The presented results highlight the importance of a detailed investigation for the specific case of application constituting the chemical system, the choice of enzyme as well as the form of immobilization. The improvement of enzymatic reaction rates by ultrasound present a first key indicator for a successful implementation of US‐ERD. In addition, there are a great number of examples where ultrasound improves the performance of chemical and electrochemical reactions.^30^, ^31^, ^35^, ^45^ Until now, the use of US in industry is limited due to inefficient US generation and high operating costs, although it is a promising alternative not solely for high‐value chemicals and pharmaceuticals.

The effects on chemical or enzymatic reactions are not yet fully understood and vary for each chemical system, since US performance is dependent on the volatility of the components.^31^ It needs to be clear whether the effects are chemical (radical formation) or physical (cavitation bubbles, mass transfer enhancement). Furthermore, equipment design, scale‐up, field uniformity, and penetration depths are crucial points in knowledge generation. Fundamental research in that area is required as accurate predictions are impossible without a fundamental understanding. Several studies deal with the theoretical description of reactor geometry, pressure field distribution and ultrasonic field propagation.^46^, ^47^ However, up to now, no study has applied these models and assembled them together in order to design an US reactor. The existing models should be validated for additional processes and a larger number of US sources to provide trustful modeling and application.

On the other hand unresolved scale‐up challenges have to be overcome in order to transfer the results from lab‐scale experiments to industrial scale, whereas maintenance of the amplitude of the acoustic power presents a main bottleneck at current state.^48^ If horns are applied as an US source their diameter has to be increased, which forces them to run at low amplitudes. A solution for this might be the Barbell Horn Ultrasonic Technology, for which amplification and equipment size are reported to be independent. Sound fields for small liquid volumes are well‐characterized and first studies for large equipment are already available.^49^, ^50^ Constructing industrial‐scale US equipment poses however further challenges concerning field generation and cavitational field distribution, for example, the installation of transducers on curved surfaces (as necessary for distillation columns) has hardly been examined.

Nevertheless, the most important issue for implementation in industry is that the impact of US has to be sufficiently large providing economic benefits.

## CONCLUSIONS

The use of US in extraction and crystallization processes enables large improvements in process rates and yields. An improvement in the extraction rate compared with batch extraction of more than 20 times was achieved for at least two processes in the ALTEREGO project. Large increases in the nucleation rate in crystallization have been shown as well, with US not needing to be continuously provided. A pulsed US operation achieves the same nucleation rate increase as the continuous US process, while at the same time decreasing the US duty by a factor of up to 10, depending on the process scale. An in‐depth analysis of US‐assisted technologies shows that the industrial potential and economic viability is still very much depending on the chemistry and type of process. A number of applications have been described here, but the library of compounds and reactions should be clearly expanded and explored, as well as the product properties that are evaluated and the knowledge on the exact moment in the process when/where US is applied.

The novel field of ultrasound‐assisted enzymatic reactive distillation (US‐ERD) provides a lot of potential that still has to be carefully evaluated for the specific case of application, taking into account the strong integration of the different aspects of this innovative technology. Basic experimental investigations have to show a positive impact of ultrasound (US) on enzymatic reaction rates and the feasibility of ERD without US has to be demonstrated. Based on a dedicated ERD model that needs to be validated with regard to experimental data, accurate predictions for process performance can be made and the validated model can be utilized for a techno‐economic evaluation of the US‐ERD process with respect to the ERD and other alternative process configurations. The presented results indicated that cost advantages are to be expected if a sufficiently high increase in enzymatic reaction rates is achieved by US application, such that the additional costs required for US equipment are overcompensated by the process improvements obtained by the use of US.

The necessary steps to a successful implementation of US‐assisted crystallization and reactive distillation in industry have further been analyzed, showing that a fundamental understanding, the development of reliable models, and appropriate scale‐up methods are of utmost importance for this purpose. While basic equipment for the implementation is already applicable, the exact construction rules and methods are yet missing and there is much room for improvement. In accordance with these challenges, key research objectives to reach within a period of ten years and a roadmap were formulated in order to foster the implementation of US‐assisted technologies in the industrial environment.
